# Maternal Serum Folic Acid Levels and Onset of Kawasaki Disease in Offspring During Infancy

**DOI:** 10.1001/jamanetworkopen.2023.49942

**Published:** 2023-12-28

**Authors:** Sayaka Fukuda, Shiro Tanaka, Chihiro Kawakami, Tohru Kobayashi, Shuichi Ito

**Affiliations:** 1 Department of Pediatrics, Graduate School of Medicine, Yokohama City University, Yokohama, Japan; 2 Department of Pediatrics, Saiseikai Yokohamashi Tobu Hospital, Yokohama, Japan; 3 Department of Clinical Biostatistics/Clinical Biostatistics Course, Graduate School of Medicine, Kyoto University, Kyoto, Japan; 4 Department of Data Science, Clinical Research Center, Hospital, National Center for Child Health and Development, Tokyo, Japan

## Abstract

**Importance:**

Kawasaki disease is an acute systemic vasculitis that primarily affects infants and young children. No reproducible risk factors have yet been identified, but a possible association between maternal folic acid supplementation and Kawasaki disease has been reported previously.

**Objective:**

To investigate the associations of exposure to maternal serum folic acid levels and maternal folic acid supplementation with onset of Kawasaki disease during infancy among offspring.

**Design, Setting, and Participants:**

This cohort study used data from the Japan Environment and Children’s Study, a nationwide birth cohort, which has enrolled children since 2011. This study used the data set released in October 2019, and analysis was performed in January 2023.

**Exposures:**

Maternal serum folic acid levels (≥10 ng/mL classified as exposed) during the second and third trimesters and the frequency of maternal folic acid supplementation during the first trimester and during the second and third trimesters of pregnancy (once a week or more was classified as exposed).

**Main Outcomes and Measures:**

The primary outcome was onset of Kawasaki disease in offspring up to age 12 months. Odds ratios (ORs) for each exposure were estimated, and propensity score–adjusted logistic regression was conducted on the basis of the sets of variables.

**Results:**

The study population comprised 87 702 children who were followed-up for 12 months. Of these, 336 children developed Kawasaki disease. Mothers who took folic acid supplements (31 275 mothers [35.7%]; mean [SD] age, 32 [5] years) had higher serum folic acid levels than those who did not take supplements. Higher maternal serum folic acid levels were associated with a significantly lower risk of Kawasaki disease in offspring than lower levels (folic acid ≥10 vs <10 ng/mL, 56 of 20 698 children [0.27%] vs 267 of 64 468 children [0.41%]; OR, 0.68; 95% CI, 0.50-0.92). Children whose mothers took folic acid supplementation during the first trimester had a lower prevalence of Kawasaki disease than children whose mothers did not take folic acid (131 of 39 098 children [0.34%] vs 203 of 48 053 children [0.42%]), although the difference was not statistically significant (OR, 0.83; 95% CI, 0.66-1.04). Supplementation during the second and third trimesters was associated with a significantly lower risk of Kawasaki disease compared with no supplementation (94 of 31 275 children [0.30%] vs 242 of 56 427 children [0.43%]; OR, 0.73; 95% CI, 0.57-0.94).

**Conclusions and Relevance:**

In this cohort study, higher serum folic acid levels (≥10 ng/mL) and maternal folic acid supplementation more than once a week during the second and third trimesters were associated with reduced risk of Kawasaki disease in offspring during infancy.

## Key Points

**Question:**

Are maternal serum folic acid levels and maternal folic acid supplementation associated with early onset of Kawasaki disease in offspring during infancy?

**Findings:**

This Japanese nationwide birth cohort study of 87 702 children found that maternal serum folic acid levels and the frequency of maternal folic acid supplementation during pregnancy were significantly associated with a decreased risk of Kawasaki disease in offspring during infancy.

**Meaning:**

These findings suggest that increasing maternal folic acid levels via maternal folic acid supplementation during pregnancy may reduce the risk of Kawasaki disease during infancy among offspring.

## Teaser

This cohort study investigates the associations of exposure to maternal serum folic acid levels and maternal folic acid supplementation with the onset of Kawasaki disease (KD) during infancy among offspring.

## Introduction

Kawasaki disease (KD) is a type of acute systemic vasculitis first described by Dr Tomisaku Kawasaki in 1967.^1^ KD primarily affects infants and young children and is the leading cause of acquired pediatric heart disease in developed countries.^2^ The reported annual incidence rate of KD in the US is 20 to 25 cases per 100 000 children younger than 5 years and has remained nearly unchanged over the last decade.^2,3,4^ Despite the declining birth rate in Japan, the number of new cases of KD continued to increase each year until 2019. The annual incidence rate of KD was 370.8 cases per 100 000 children younger than 5 years in 2019 but decreased to 238.8 cases per 100 000 in 2020.^5^ This is thought to be owing to a sudden decrease in epidemic infections such as influenza and respiratory syncytial virus as a result of infection prevention behavior during the COVID-19 pandemic.^6^ A decrease in new KD cases during 2020 was similarly observed in many countries worldwide.^7,8^ Although the cause of KD remains unknown, this phenomenon indicates that several infectious agents may be involved. However, the impact of infection on the mechanism by which KD develops may be limited because the prevalence of various infectious diseases does not coincide with trends in the number of KD cases. In Japan, patients younger than 1 year account for approximately 20% of all patients with KD.^5^ A high risk of KD during infancy suggests that some type of exposure in utero may be associated with the onset of the disease. Previous epidemiologic studies have reported associations between KD onset and prenatal exposures such as maternal age,^9^ maternal autoimmune disease,^10^ and maternal smoking.^11^ However, to date, no exposure has been identified that has a reproducible association with the onset of KD; our previous studies also did not reveal any such association.^12^

In our previous study,^12^ we reported a correlation between folic acid supplementation during pregnancy and the risk of KD in a large birth cohort in Japan. In the present study, we aimed to confirm those findings using confounder adjustment based on literature reviews of risk factors for KD and propensity scores, as well as data on serum folic acid levels in pregnant women.

## Methods

### Study Design, Settings, and Participants

In this cohort study, we used data from the Japan Environment and Children’s Study (JECS), a large Japanese prospective birth cohort.^13,14^ Participants were recruited across the 15 regional centers from January 2011 to March 2014. The JECS has been performed in accordance with the Declaration of Helsinki^15^ and the Japanese Ethical Guidelines for Epidemiological Research published by the Ministry of Education, Culture, Sports, Science and Technology and the Ministry of Health, Labour and Welfare, Japan.^16^ The JECS protocol was reviewed and approved by the Ministry of the Environment’s Institutional Review Board on Epidemiological Studies and the Ethics Committees of all participating institutions.^13^ All participants provided written informed consent. We followed the Strengthening the Reporting of Observational Studies in Epidemiology (STROBE) reporting guidelines for cohort studies.

The present study is based on the jecs-ta-2019030-qsn data set, which was released in October 2019; we analyzed this data set in January 2023. We excluded fetuses who were miscarried or stillborn and those lost to follow-up after birth. We excluded live-born children who met the following exclusion criteria: (1) no available data on maternal folic acid supplementation during pregnancy, (2) the parents did not complete the questionnaires at both the child’s age 6 months and 1 year, and (3) the parents stated that their child had no history of KD at 6 months but did not respond to the survey when their child was 1 year old.

### Variables

#### Outcome

The primary outcome was the onset of KD in infancy (up to age 12 months). We defined children with KD (KD group) as those whose mothers reported that their child had a history of KD on the questionnaire at age 6 months and/or 1 year. KD was diagnosed according to the *Revision of Diagnostic Guidelines for Kawasaki Disease (5th Revised Edition)*, which was applicable at the time.^17^ All other participants were classified as not having KD (non-KD group).

#### Exposures

We obtained maternal serum folic acid levels in nanograms per milliliter (to convert folic acid to nanomoles per liter, multiply by 2.266) from blood sampling data collected during the second and third trimesters of pregnancy as the primary exposure. As secondary exposures, we obtained data from mothers using self-administered questionnaires regarding the frequency of maternal folic acid supplementation in the first trimester and during the second and third trimesters.

#### Other Variables

We chose variables of interest in association with KD that were related to parental characteristics, prenatal and perinatal backgrounds, and children’s characteristics using self-administered questionnaires completed by mothers during the first trimester, during the second and third trimesters, at 1 month, 6 months, and at 1 year after birth; from fathers at registration; and from the medical records during the first trimester, at birth, and at 1 month after birth. Parental characteristics included the following variables: maternal prepregnancy physical characteristics (height, weight, and body mass index [calculated as weight in kilograms divided by height in meters squared]), maternal medical history (KD, allergic diseases, neurological or psychiatric diseases, diabetes, and cancer), paternal medical history (KD and allergic diseases), and parental socioeconomic status (maternal and paternal education and household income).

Regarding prenatal and perinatal background, the following variables were included: the method of pregnancy (spontaneous or artificial), maternal complications during the pregnancy (thyroid disease and diabetes), maternal dietary intake (beans [grams per day], green and yellow vegetables [grams per day], fruits [grams per day], fermented foods [cheese, yogurt, nattō, or *Lactobacillus* species–fermented beverages; grams per day], estimated folic acid from daily dietary intake [micrograms per day]),^18^ use of supplements other than folic acid during the second and third trimesters (zinc, eicosapentaenoic acid, docosahexaenoic acid, and *Lactobacillus* species–fermented beverages), maternal smoking and drinking, maternal age at the child’s birth, perinatal complications (threatened abortion or premature labor, gestational diabetes, gestational hypertension, and premature membrane rupture), and mode of delivery (cesarean delivery). Children’s characteristics included preterm birth (≤36 weeks), sex of the child (male), birth weight, neonatal jaundice requiring treatment, nutritional methods (breastfeeding only), body weight gain per day up to age 1 month, and presence of siblings (for the child).

We dichotomized parental educational history as beyond high school graduate or not and annual household income as greater than or equal to ¥4 million vs less than ¥4 million (as of November 27, 2023, ¥1 = US $0.067). We defined maternal smoking or drinking if mothers reported smoking or drinking at either or both time points (in the first trimester and the second and third trimesters). Supplementation of zinc, eicosapentaenoic acid, docosahexaenoic acid, and *Lactobacillus* species–fermented beverages was dichotomized according to whether these were taken more than once a month.

### Statistical Analysis

Exposure variables were binarized in the statistical analysis, and the cutoff values were determined according to the distribution of maternal serum folic acid levels, the frequency of folic acid supplementation, and clinical considerations. The primary exposure variable was defined using the third and fourth quartiles (≥10 ng/mL) of maternal serum folic acid levels as exposed and the first and second quartiles as not exposed. We also conducted sensitivity analysis using 3 exposure levels (<10, 10-19, and ≥20 ng/mL). The frequency of folic acid supplementation was measured using 4 categories: daily, at least once a week, at least once a month, and never. Children with maternal intake of folic acid supplementation once a week or more were classified as exposed; otherwise, children were classified as not exposed.

We estimated odds ratios (ORs) and 95% CIs for the associations between each of 3 exposures (maternal serum folic acid level of ≥10 ng/mL, folic acid supplementation more than once a week during the first trimester, and folic acid supplementation more than once a week during the second and third trimesters) and onset of KD in children using logistic regression adjusted for a linear term of propensity score. For each child, we calculated the propensity score, defined as the conditional probability of a child being exposed given the covariates, using logistic regression. We fitted a total of 9 logistic regression models to combinations of the 3 exposures and 3 sets of covariates. We constructed 3 models including different potential confounders; for each model, we calculated the propensity score using logistic regression analysis with KD onset as the outcome variable. The variables for each model were selected according to the following criteria. Model 1 (main analysis) included all variables, excluding postnatal variables (see eTables 1, 2, and 3 in Supplement 1 for details). In model 2 (sensitivity analysis), we selected only 9 variables that may be associated with the development of KD (eTable 4 in Supplement 1). Model 3 (sensitivity analysis) included only 8 variables associated with folic acid supplementation behavior (eTable 5 in Supplement 1). Furthermore, in each model, when analyzing the association with maternal serum folic acid levels, we excluded variables that were assumed to have a direct effect on serum folic acid levels (ie, frequency of folic acid supplementation and maternal dietary intake). Similarly, we excluded maternal serum folic acid levels and frequency of folic acid supplementation during different gestational periods in analysis of the association with frequency of folic acid supplementation. Using the missing-indicator method, we created an extra indicator variable to identify whether the value for that covariate was missing; missing values were replaced with one single value (eg, with the value 0). The positivity assumption was examined using a histogram of the calculated propensity scores. We calculated the propensity score for each child using the covariates in model 1, dichotomized according to the median maternal serum folic acid level. We excluded 1777 children with a propensity score below the first centile and above the 99th centile (eFigure 1 in Supplement 1). The potential for an unmeasured confounder was assessed using E-values. An academic statistician (S.T.) conducted all analyses using SAS statistical software version 9.4 (SAS Institute).

## Results

**Figure 1.**
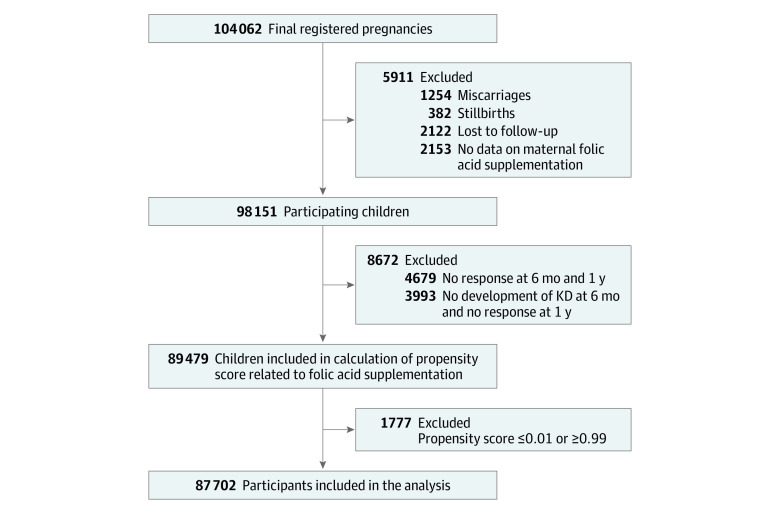
Flowchart of Participant Selection KD indicates Kawasaki disease.

Figure 1 shows the flowchart of participant selection in this study. A total of 104 062 fetal records were registered during the period of the survey. We excluded participants according to our criteria. Finally, 87 702 participants were included in the analysis; 31 275 mothers (35.7%; mean [SD] age, 32 [5] years) took folic acid supplements. A total of 336 children developed KD by age 1 year. The background of the excluded participants is shown in eTable 6 in Supplement 1.

Table 1 presents the characteristics and backgrounds of participants in the KD group and non-KD group. In both groups, the estimated folic acid intake from meals was 260 μg per day. The backgrounds of participants were also compared according to each exposure. In a comparison between maternal serum folic acid level greater than or equal to 10 ng/mL and less than 10 ng/mL, the group with higher maternal serum folic acid levels was more likely to have higher socioeconomic status, a higher percentage of supplementation including folic acid, older mothers, and a higher proportion of children with no siblings (eTable 7 in Supplement 1). In addition, in a comparison between maternal folic acid supplementation and no supplementation during the first and during the second and third trimesters, the group with supplementation was more likely to have higher socioeconomic status, a higher percentage of other supplementation, higher maternal serum folic acid levels, older mothers, and a higher proportion of children with no siblings (eTables 8 and 9 in Supplement 1).

**Table 1.** Characteristics and Background of Participants Included in the Analysis

Variables	Participants, No./total No. (%)	
	KD group (n = 336)	Non-KD group (n = 87 366)
Maternal prepregnancy physical characteristics		
Height, cm		
Mean (SD)	158.6 (5.3)	158.1 (5.3)
Median (range)	158.0 (143.0 to 173.0)	158.0 (130.0 to 183.0)
Weight before pregnancy, kg		
Mean (SD)	52.9 (8.8)	52.9 (8.6)
Median (range)	51.0 (39.0 to 95.0)	52.0 (29.0 to 127.6)
Body mass index before pregnancy^a^		
Mean (SD)	21.0 (3.3)	21.2 (3.2)
Median (range)	20.1 (15.4 to 41.7)	20.5 (13.2 to 52.8)
Maternal medical history		
KD	1/335 (0.3)	377/87 037 (0.4)
Allergic disease	178/335 (53.1)	48 388/87 037 (55.6)
Neurological or psychiatric disease	47/335 (14.0)	11 802/87 037 (13.6)
Diabetes	4/335 (1.2)	816/87 037 (0.9)
Cancer	7/335 (2.1)	935/87 037 (1.1)
Paternal medical history		
KD	2/149 (1.3)	196/45 432 (0.4)
Allergic disease	84/149 (56.4)	21 112/45 432 (46.5)
Parental socioeconomic status		
Maternal education beyond high school graduate	221/335 (66.0)	56 990/87 040 (65.5)
Paternal education beyond high school graduate	193/333 (58.0)	49 580/86 581 (57.3)
Annual household income ≥¥4 million^b^	200/315 (63.5)	49 600/81 599 (60.8)
Spontaneous pregnancy	305/336 (90.8)	81 007/87 342 (92.7)
Pregnancy complications		
Thyroid disease	9/336 (2.7)	1169/87 199 (1.3)
Diabetes	1/336 (0.3)	905/87 199 (1.0)
Dietary intake during the second and third trimesters		
Beans, g/d		
Mean (SD)	59 (144)	54 (75)
Median (range)	36 (0 to 2491)	34 (0 to 3960)
Vegetables, g/d		
Mean (SD)	82 (78)	89 (92)
Median (range)	64 (0 to 828)	66 (0 to 4705)
Fruits, g/d		
Mean (SD)	154 (156)	146 (167)
Median (range)	117 (0 to 1275)	108 (0 to 9468)
Fermented foods, g/d		
Mean (SD)	116 (141)	122 (158)
Median (range)	84 (0 to 1153)	84 (0 to 3171)
Estimated folic acid from daily meals, μg/d		
Mean (SD)	260 (161)	260 (162)
Median (range)	223 (17 to 1411)	230 (3 to 9253)
Supplementation during the second and third trimesters		
Folic acid		
Daily	52/336 (15.5)	18 872/87 366 (21.6)
At least once a week	42/336 (12.5)	12 309/87 366 (14.1)
At least once a month	28/336 (8.3)	6507/87 366 (7.4)
Never	214/336 (63.7)	49 678/87 366 (56.9)
Zinc	7/333 (2.1)	3369/87 039 (3.9)
Eicosapentaenoic acid	5/334 (1.5)	983/86 923 (1.1)
Docosahexaenoic acid	7/335 (2.1)	1930/86 972 (2.2)
*Lactobacillus* species–fermented beverages	173/336 (51.5)	46 015/87 161 (52.8)
Lifestyle habits		
Smoking during pregnancy	15/331 (4.5)	3633/86 089 (4.2)
Drinking during pregnancy	40/330 (12.1)	9732/86 158 (11.3)
Maternal serum folic acid level during the second and third trimesters, ng/mL		
Mean (SD)	6.9 (4.4)	7.7 (4.9)
Median (range)	5.3 (1.6 to 20.0)	6.0 (0.7 to 20.0)
Maternal age at birth, y		
Mean (SD)	31 (5)	31 (5)
Median (range)	31 (20 to 45)	31 (14 to 49)
Perinatal complications		
Threatened abortion or premature labor	95/336 (28.3)	23 603/87 199 (27.1)
Gestational diabetes	5/336 (1.5)	2336/87 199 (2.7)
Gestational hypertension	11/336 (3.3)	2702/87 199 (3.1)
Premature rupture of membranes	35/336 (10.4)	7231/87 199 (8.3)
Cesarean delivery	59/335 (17.6)	17 105/86 995 (19.7)
Child characteristics		
Preterm birth (≤36 wk)	14/336 (4.2)	4563/87 199 (5.2)
Male sex	185/336 (55.1)	44 813/87 366 (51.3)
Birth weight, g		
Mean (SD)	3024 (407)	3014 (423)
Median (range)	3020 (914 to 4304)	3022 (398 to 5214)
Neonatal jaundice with treatment	53/331 (16.0)	13 236/84 799 (15.6)
Breastfeeding only	138/333 (41.4)	37 045/86 956 (42.6)
Weight gain per day, g		
Mean (SD)	39 (11)	39 (12)
Median (range)	39 (3 to 74)	39 (−9 to 89)
Presence of child’s siblings	219/335 (65.4)	50 561/87 037 (58.1)

Abbreviation: KD, Kawasaki disease.

SI conversion factor: To convert folic acid to nanomoles per liter, multiply by 2.266.

^a^ Body mass index is calculated as weight in kilograms divided by height in meters squared.

^b^ As of November 27, 2023, ¥1 = US $0.067.

**Figure 2.**
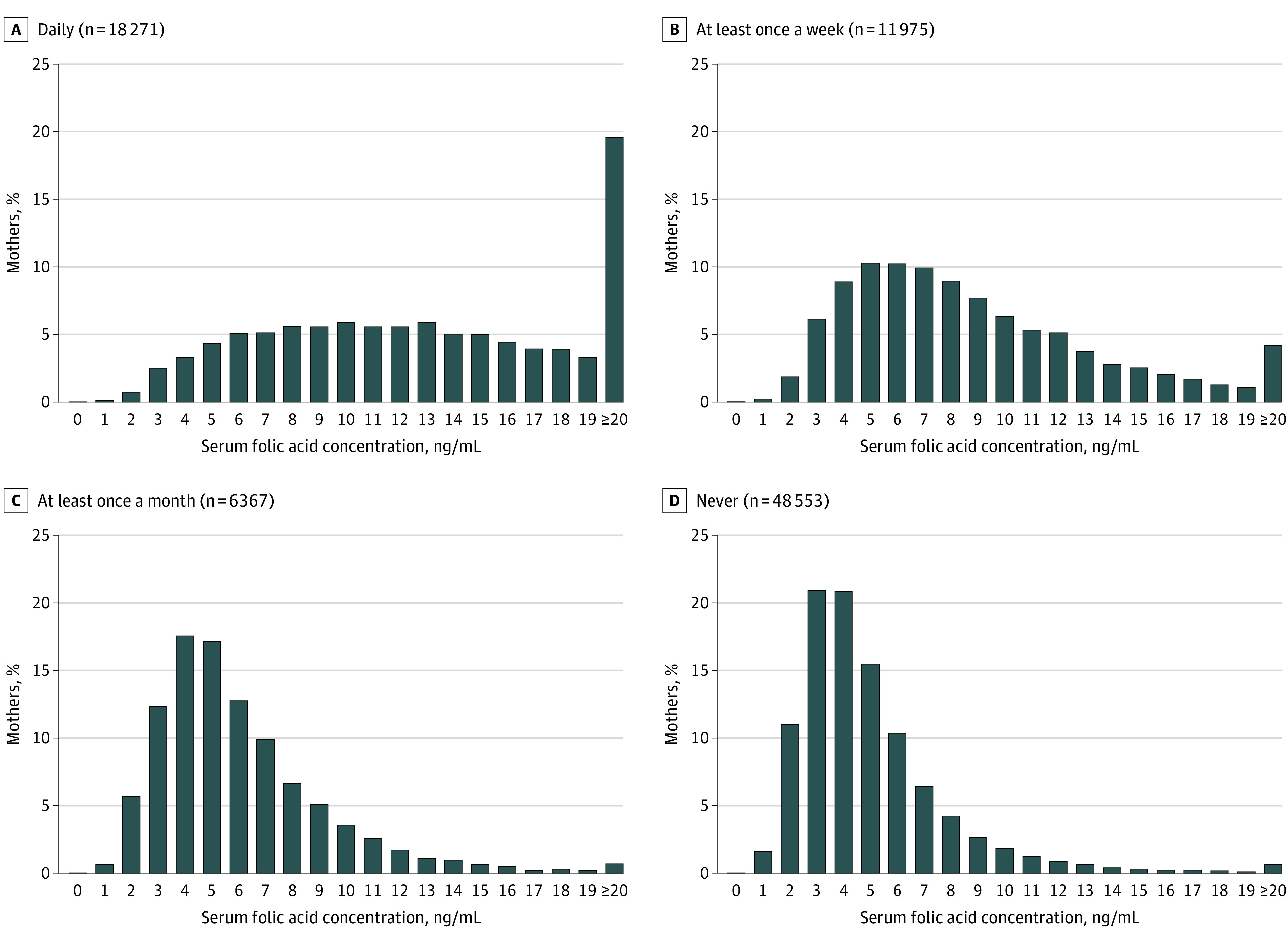
Distribution of Maternal Serum Folic Acid Levels by Frequency of Folic Acid Supplementation Graphs show serum folic acid concentration for maternal supplementation received daily (A), at least once a week (B), at least once a month (C), and never (D). To convert folic acid to nanomoles per liter, multiply by 2.266.

Figure 2 shows the distribution of maternal serum folic acid levels by frequency of folic acid supplementation. We further divided mothers into 4 groups according to serum folic acid levels and examined the frequency of folic acid supplementation in each group. We found that groups with higher serum levels had a higher proportion of mothers who took supplements more frequently (eFigure 2 in Supplement 1).

Table 2 shows the results of propensity score analysis of the association between maternal serum folic acid levels in the second and third trimesters and onset of KD. The rates of KD in the groups with higher (≥10 ng/mL) and lower (<10 ng/mL) maternal serum folic acid levels were 56 of 20 698 children (0.27%) and 267 of 64 468 children (0.41%), respectively. Maternal folic acid levels were significantly associated with a decreased risk of KD in model 1 (OR, 0.68; 95% CI 0.50-0.92). The other 2 models also showed significant associations of folic acid supplementation with KD. We also conducted sensitivity analysis using 3 folic acid exposure levels (<10, 10-19, and ≥20 ng/mL) (eTable 10 in Supplement 1). The results showed that in all 3 models, only the 10 to 19 ng/mL group had a significantly decreased incidence of KD. In terms of the actual incidence of KD, the 10 to 19 ng/mL group and greater than or equal to 20 ng/mL group were comparable.

**Table 2.** Propensity Score Analysis of Maternal Serum Folic Acid Levels During the Second and Third Trimesters and Onset of KD

Model and serum folic acid level	Development of KD, No. of patients/total No. (%)	OR (95% CI)	E-value
Model 1^a^			
<10 ng/mL	267/64 468 (0.41)	1 [Reference]	2.30
≥10 ng/mL	56/20 698 (0.27)	0.68 (0.50-0.92)	
Model 2^b^			
<10 ng/mL	267/64 468 (0.41)	1 [Reference]	2.30
≥10 ng/mL	56/20 698 (0.27)	0.68 (0.51-0.91)	
Model 3^c^			
<10 ng/mL	267/64 468 (0.41)	1 [Reference]	2.26
≥10 ng/mL	56/20 698 (0.27)	0.69 (0.51-0.92)	

Abbreviations: KD, Kawasaki disease; OR, odds ratio.

SI conversion factor: To convert folic acid to nanomoles per liter, multiply by 2.266.

^a^ Details of covariates included in model 1 are presented in eTable 1 in Supplement 1.

^b^ Details of covariates included in model 2 are presented in eTable 4 in Supplement 1.

^c^ Details of covariates included in model 3 are presented in eTable 5 in Supplement 1.

Table 3 shows the results of propensity score analysis of the association of maternal folic acid supplementation with the onset of KD. Children whose mothers took folic acid supplementation during the first trimester had a lower prevalence of KD vs children whose mothers did not take folic acid (131 of 39 098 children [0.34%] vs 203 of 48 053 children [0.42%]), although the difference was not statistically significant (OR, 0.83; 95% CI, 0.66-1.04) in model 1, in which postnatal variables were excluded. The other 2 models with limited variables had results similar to those of model 1. Similarly, the rates of KD in the groups with and without supplementation during the second and third trimesters were 94 of 31 275 children (0.30%) and 242 of 56 427 children (0.43%), respectively. During the second and third trimesters, model 1 showed significant associations of folic acid supplementation with a decreased risk of KD (OR, 0.73; 95% CI, 0.57-0.94). The other 2 models also showed significant associations with KD.

**Table 3.** Propensity Score Analysis of Maternal Folic Acid Supplementation and Onset of KD

Exposure and model	Development of KD, No. of patients/total No. (%)	OR (95% CI)	E-value
Folic acid supplementation during the first trimester			
Model 1^a^			
No	203/48 053 (0.42)	1 [Reference]	1.70
Yes	131/39 098 (0.34)	0.83 (0.66-1.04)	
Model 2^b^			
No	203/48 053 (0.42)	1 [Reference]	1.77
Yes	131/39 098 (0.34)	0.81 (0.65-1.02)	
Model 3^c^			
No	203/48 053 (0.42)	1 [Reference]	1.70
Yes	131/39 098 (0.34)	0.83 (0.66-1.04)	
Folic acid supplementation during the second and third trimesters			
Model 1^d^			
No	242/56 427 (0.43)	1 [Reference]	2.08
Yes	94/31 275 (0.30)	0.73 (0.57-0.94)	
Model 2^b^			
No	242/56 427 (0.43)	1 [Reference]	2.12
Yes	94/31 275 (0.30)	0.72 (0.56-0.91)	
Model 3^c^			
No	242/56 427 (0.43)	1 [Reference]	2.08
Yes	94/31 275 (0.30)	0.73 (0.57-0.94)	

Abbreviations: KD, Kawasaki disease; OR, odds ratio.

^a^ Details of covariates included in model 1 are presented in eTable 2 in Supplement 1.

^b^ Details of covariates included in model 2 are presented in eTable 4 in Supplement 1.

^c^ Details of covariates included in model 3 are presented in eTable 5 in Supplement 1.

^d^ Details of covariates included in model 1 are presented in eTable 3 in Supplement 1.

## Discussion

In this cohort study, we found that higher maternal serum folic acid levels in the second and third trimesters of pregnancy were significantly associated with a lower risk of KD onset among offspring during infancy. Similarly, infants born to mothers who more frequently took folic acid supplements in the second and third trimesters of pregnancy had a lower risk of KD in infancy. In contrast, the associations with estimated maternal daily dietary intake of folic acid were similar in the KD group and non-KD group. These facts suggest that maternal folic acid supplementation during pregnancy may reduce the risk of KD in offspring during infancy.

We examined the association of maternal folic acid intake with the onset of KD in offspring from an epidemiological perspective. As the number of new cases of KD in Japan has progressively increased,^5^ folic acid intake during pregnancy among Japanese women has decreased. According to a national survey, dietary intake of folic acid among Japanese pregnant women is declining, down approximately 20% from the rate 20 years earlier.^19^ A report^20^ from a national survey by the Ministry of Health, Labour and Welfare in 2019 also showed that dietary folic acid intake among pregnant women was only 243 μg per day. However, the World Health Organization recommendation for dietary folic acid intake is at least 480 μg per day.^20^ The results of our study showed that the average folic acid intake in both the KD and control groups was 260 μg per day. In addition, although results vary widely depending on the survey population and methods, the percentage of Japanese pregnant women who take folic acid supplements from preconception is only approximately 20%, which is insufficient.^21,22^ In the current study, more than 60% of pregnant women did not take any folic acid supplements at all. Another important point is that folic acid intake through supplementation is more efficient in terms of bioavailability compared with intake through the diet.^23,24^ Our results also showed that daily folic acid supplementation in addition to daily dietary intake was associated with increased maternal serum folic acid levels. On the basis of these findings, we speculate that taking additional folic acid supplements with an appropriate diet during pregnancy may reduce the risk of KD in offspring.

We considered the pathophysiological mechanisms of how maternal folic acid supplementation could reduce the risk of KD development in the offspring. To our knowledge, there are currently no studies demonstrating a direct association between maternal folic acid supplementation during pregnancy and KD. Because it is known that certain immunological mechanisms are involved in the pathogenesis of KD, folic acid may have an effect on the immune system. For example, folic acid inhibits inflammation by suppressing cytokines and chemokines from inflammatory cells or by maintaining lymphocyte function.^25,26,27,28^ Conversely, in a folic acid–deficient culture, an enhanced inflammatory response to lipopolysaccharide in murine macrophages, impaired dendritic cell function, and subsequent T-helper cell differentiation have been observed.^27,29^ In addition, chronic folic acid deficiency reportedly increases the production of monocyte chemoattractant protein–1 from human vascular endothelial cells and induces more intense inflammation.^30^

DNA methylation is an epigenetic modification essential for genome regulation and development. Folic acid is a key component in DNA methylation.^31^ Maternal serum folic acid levels are positively correlated with folic acid and its metabolites in cord blood.^32,33,34^ Maternal folic acid intake during pregnancy can affect not only the maternal immune system but also DNA methylation in the offspring.^35,36^ Furthermore, levels of folic acid and its metabolites in cord blood are associated with epigenetic changes in the immune cells of offspring, indicating that epigenetic mechanisms can affect the offspring across generations.^37,38,39^ On the basis of the aforementioned studies, maternal serum folic acid levels may lead to epigenetic changes in immune cells, which, in turn, lead to differences in the risk of KD among offspring. However, to prove this concept, prospective randomized clinical trials are necessary to clarify the causal relationship between maternal folic acid supplementation during pregnancy and the risk of KD in offspring.

### Limitations

Our study has several limitations. Because we examined the onset of KD among infants up to age 12 months only, further studies are needed to extend the observation period. In addition, we were unable to evaluate the effect of folic acid alone because the supplements taken by participating mothers may have been complex supplements, such as multivitamins, rather than supplements containing folic acid alone. There is also unmeasured confounding that was not adjusted in the propensity score analysis.

## Conclusions

In this study, we examined the association of exposure to maternal folic acid supplementation with early onset of KD among offspring in relation to maternal serum folic acid levels during pregnancy, using propensity scores. Through increasing maternal serum folic acid levels, maternal folic acid supplementation during the second and third trimesters of pregnancy may reduce the cumulative incidence of early-onset KD by the infant’s age 1 year.

## Notes

Supplement 1. **eTable 1.** List of Covariates Included in Model 1 in the Analysis With Exposure of Maternal Serum Folic Acid Levels **eTable 2.** List of Covariates Included in Model 1 in the Analysis With Exposure of Folic Acid Supplementation During the First Trimester **eTable 3.** List of Covariates Included in Model 1 in the Analysis With Exposure of Folic Acid Supplementation During the Second/Third Trimester **eTable 4.** List of Covariates Included in Model 2 **eTable 5.** List of Covariates Included in Model 3 **eTable 6.** Characteristics and Background of Participants Included and Excluded in the Analysis^a^ **eTable 7.** Characteristics and Background of Participants (Maternal Serum Folic Acid Level ≥10 ng/mL or <10 ng/mL)^a^ **eTable 8.** Characteristics and Background of Participants (With and Without Maternal Folic Acid Supplementation During the First Trimester)^a^ **eTable 9.** Characteristics and Background of Participants (With and Without Maternal Folic Acid Supplementation During the Second/Third Trimester)^a^ **eTable 10.** Propensity Score Analysis of Maternal Serum Folic Acid Levels During the Second/Third Trimester and Onset of Kawasaki Disease^a^ **eFigure 1.** Distribution of Propensity Scores Based on Model 1 According to Maternal Serum Folic Acid Levels **eFigure 2.** Frequency of Folic Acid Supplementation According to Serum Folic Acid Level

Supplement 2.
Japan Environment and Children’s Study Group Members

Supplement 3.
Data Sharing Statement
