# Supplementary material for: Maternal Serum Folic Acid Levels and Onset of Kawasaki Disease in Offspring During Infancy
Source: JAMA Netw Open. 2023 Dec 28;6(12):e2349942. doi: 10.1001/jamanetworkopen.2023.49942 (PMC10755611; doi:10.1001/jamanetworkopen.2023.49942)
Supplement: Supplement 1. — eTable 1. List of Covariates Included in Model 1 in the Analysis With Exposure of Maternal Serum Folic Acid Levels eTable 2. List of Covariates Included in Model 1 in the Analysis With Exposure of Folic Acid Supplementation During the First Trimester eTable 3. List of Covariates Included in Model 1 in the Analysis With Exposure of Folic Acid Supplementation During the Second/Third Trimester eTable 4. List of Covariates Included in Model 2 eTable 5. List of Covariates Included in Model 3 eTable 6. Characteristics and Background of Participants Included and Excluded in the Analysisa eTable 7. Characteristics and Background of Participants (Maternal Serum Folic Acid Level ≥10 ng/mL or <10 ng/mL)a eTable 8. Characteristics and Background of Participants (With and Without Maternal Folic Acid Supplementation During the First Trimester)a eTable 9. Characteristics and Background of Participants (With and Without Maternal Folic Acid Supplementation During the Second/Third Trimester)a eTable 10. Propensity Score Analysis of Maternal Serum Folic Acid Levels During the Second/Third Trimester and Onset of Kawasaki Diseasea eFigure 1. Distribution of Propensity Scores Based on Model 1 According to Maternal Serum Folic Acid Levels eFigure 2. Frequency of Folic Acid Supplementation According to Serum Folic Acid Level [file jamanetwopen-e2349942-s001.pdf]

Supplemental Online Content

Fukuda S, Tanaka S, Kawakami C, Kobayashi T, Ito S; Japan Environment and Children's Study Group. Maternal serum folic acid levels and onset of Kawasaki disease in offspring during infancy. *JAMA Netw Open*. 2024;7(1):e2349942.
doi:10.1001/jamanetworkopen.2023.49942

eTable 1. List of Covariates Included in Model 1 in the Analysis With Exposure of Maternal Serum Folic Acid Levels
eTable 2. List of Covariates Included in Model 1 in the Analysis With Exposure of Folic Acid Supplementation During the First Trimester
eTable 3. List of Covariates Included in Model 1 in the Analysis With Exposure of Folic Acid Supplementation During the Second/Third Trimester
eTable 4. List of Covariates Included in Model 2
eTable 5. List of Covariates Included in Model 3
eTable 6. Characteristics and Background of Participants Included and Excluded in the Analysis[a]
eTable 7. Characteristics and Background of Participants (Maternal Serum Folic Acid Level ≥10 ng/mL or <10 ng/mL)[a]
eTable 8. Characteristics and Background of Participants (With and Without Maternal Folic Acid Supplementation During the First Trimester)[a]
eTable 9. Characteristics and Background of Participants (With and Without Maternal Folic Acid Supplementation During the Second/Third Trimester)[a]
eTable 10. Propensity Score Analysis of Maternal Serum Folic Acid Levels During the Second/Third Trimester and Onset of Kawasaki Disease[a]
eFigure 1. Distribution of Propensity Scores Based on Model 1 According to Maternal Serum Folic Acid Levels
eFigure 2. Frequency of Folic Acid Supplementation According to Serum Folic Acid Level

This supplemental material has been provided by the authors to give readers additional information about their work.

© 2024 Fukuda S et al. *JAMA Network Open.*

eTable 1. List of Covariates Included in Model 1 in the Analysis With Exposure of Maternal Serum Folic Acid Levels

Covariates	Included in the model
Maternal pre-pregnancy physical characteristics	
Height	✓
Weight before pregnancy	✓
Body mass index before pregnancy	✓
Maternal medical history	
Kawasaki disease	✓
Allergic disease	✓
Neurological/psychiatric disease	✓
Diabetes mellitus	✓
Cancer	✓
Paternal medical history	
Kawasaki disease	✓
Allergic disease	✓
Parental socioeconomic status	
Maternal education	✓
Paternal education	✓
Household income	✓
Spontaneous pregnancy	✓
Pregnancy complications	
Thyroid disease	✓
Diabetes mellitus	✓
Dietary intake during the second/third trimester	
Beans	
Vegetables	
Fruits	
Fermented foods	
Estimated folic acid from daily meals	

© 2024 Fukuda S et al. *JAMA Network Open.*

eTable 1. List of Covariates Included in Model 1 in the Analysis With Exposure of Maternal Serum Folic Acid Levels (continued)

Covariates	Included in the model
Supplementation during the first trimester (folic acid)	
Supplementation during the second/third trimester	
Folic acid	
Zinc	✓
Eicosapentaenoic acid	✓
Docosahexaenoic acid	✓
Lactobacillus-fermented beverages	✓
Lifestyle habits	
Smoking during pregnancy	✓
Drinking during pregnancy	✓
Maternal serum folic acid level during the second/third trimester	
Maternal age at birth	✓
Perinatal complications	
Threatened abortion/premature labor	✓
Gestational diabetes mellitus	✓
Gestational hypertension	✓
Premature rupture of membranes	✓
Mode of delivery (cesarean delivery)	✓
Child characteristics	
Preterm birth (≤36 weeks)	
Male sex	✓
Birth weight	
Neonatal jaundice with treatment	
Breastfeeding only	
Weight gain per day	
Presence of child's siblings	✓

© 2024 Fukuda S et al. *JAMA Network Open.*

eTable 2. List of Covariates Included in Model 1 in the Analysis With Exposure of Folic Acid Supplementation During the First Trimester

Covariates	Included in the model
Maternal pre-pregnancy physical characteristics	
Height	✓
Weight before pregnancy	✓
Body mass index before pregnancy	✓
Maternal medical history	
Kawasaki disease	✓
Allergic disease	✓
Neurological/psychiatric disease	✓
Diabetes mellitus	✓
Cancer	✓
Paternal medical history	
Kawasaki disease	✓
Allergic disease	✓
Parental socioeconomic status	
Maternal education	✓
Paternal education	✓
Household income	✓
Spontaneous pregnancy	✓
Pregnancy complications	
Thyroid disease	✓
Diabetes mellitus	✓
Dietary intake during the second/third trimester	
Beans	✓
Vegetables	✓
Fruits	✓
Fermented foods	✓
Estimated folic acid from daily meals	✓

© 2024 Fukuda S et al. *JAMA Network Open.*

eTable 2. List of Covariates Included in Model 1 in the Analysis With Exposure of Folic Acid Supplementation During the First Trimester (continued)

Covariates	Included in the model
Supplementation during the first trimester (folic acid)	
Supplementation during the second/third trimester	
Folic acid	
Zinc	✓
Eicosapentaenoic acid	✓
Docosahexaenoic acid	✓
Lactobacillus-fermented beverages	✓
Lifestyle habits	
Smoking during pregnancy	✓
Drinking during pregnancy	✓
Maternal serum folic acid level during the second/third trimester	
Maternal age at birth	✓
Perinatal complications	
Threatened abortion/premature labor	✓
Gestational diabetes mellitus	✓
Gestational hypertension	✓
Premature rupture of membranes	✓
Mode of delivery (cesarean delivery)	✓
Child characteristics	
Preterm birth (≤36 weeks)	
Male sex	✓
Birth weight	
Neonatal jaundice with treatment	
Breastfeeding only	
Weight gain per day	
Presence of child's siblings	✓

© 2024 Fukuda S et al. *JAMA Network Open.*

eTable 3. List of Covariates Included in Model 1 in the Analysis With Exposure of Folic Acid Supplementation During the Second/Third Trimester

Covariates	Included in the model
Maternal pre-pregnancy physical characteristics	
Height	✓
Weight before pregnancy	✓
Body mass index before pregnancy	✓
Maternal medical history	
Kawasaki disease	✓
Allergic disease	✓
Neurological/psychiatric disease	✓
Diabetes mellitus	✓
Cancer	✓
Paternal medical history	
Kawasaki disease	✓
Allergic disease	✓
Parental socioeconomic status	
Maternal education	✓
Paternal education	✓
Household income	✓
Spontaneous pregnancy	✓
Pregnancy complications	
Thyroid disease	✓
Diabetes mellitus	✓
Dietary intake during the second/third trimester	
Beans	✓
Vegetables	✓
Fruits	✓
Fermented foods	✓
Estimated folic acid from daily meals	✓

© 2024 Fukuda S et al. *JAMA Network Open.*

eTable 3. eTable 3. List of Covariates Included in Model 1 in the Analysis With Exposure of Folic Acid Supplementation During the Second/Third Trimester (continued)

Covariates	Included in the model
Supplementation during the first trimester (folic acid)	
Supplementation during the second/third trimester	
Folic acid	
Zinc	✓
Eicosapentaenoic acid	✓
Docosahexaenoic acid	✓
Lactobacillus-fermented beverages	✓
Lifestyle habits	
Smoking during pregnancy	✓
Drinking during pregnancy	✓
Maternal serum folic acid level during the second/third trimester	
Maternal age at birth	✓
Perinatal complications	
Threatened abortion/premature labor	✓
Gestational diabetes mellitus	✓
Gestational hypertension	✓
Premature rupture of membranes	✓
Mode of delivery (cesarean delivery)	✓
Child characteristics	
Preterm birth (≤36 weeks)	
Male sex	✓
Birth weight	
Neonatal jaundice with treatment	
Breastfeeding only	
Weight gain per day	
Presence of child's siblings	✓

© 2024 Fukuda S et al. *JAMA Network Open.*

eTable 4. List of Covariates Included in Model 2

Covariates	Included in the model
Maternal pre-pregnancy physical characteristics	
Height	
Weight before pregnancy	
Body mass index before pregnancy	
Maternal medical history	
Kawasaki disease	✓
Allergic disease	
Neurological/psychiatric disease	
Diabetes mellitus	
Cancer	
Paternal medical history	
Kawasaki disease	✓
Allergic disease	
Parental socioeconomic status	
Maternal education	✓
Paternal education	✓
Household income	✓
Spontaneous pregnancy	
Pregnancy complications	
Thyroid disease	✓
Diabetes mellitus	
Dietary intake during the second/third trimester	
Beans	
Vegetables	
Fruits	
Fermented foods	
Estimated folic acid from daily meals	

© 2024 Fukuda S et al. *JAMA Network Open.*

eTable 4. List of Covariates Included in Model 2 (continued)

Covariates	Included in the model
Supplementation during the first trimester (folic acid)	
Supplementation during the second/third trimester	
Folic acid	
Zinc	
Eicosapentaenoic acid	
Docosahexaenoic acid	
Lactobacillus-fermented beverages	
Lifestyle habits	
Smoking during pregnancy	✓
Drinking during pregnancy	
Maternal serum folic acid level during the second/third trimester	
Maternal age at birth	
Perinatal complications	
Threatened abortion/premature labor	
Gestational diabetes mellitus	
Gestational hypertension	
Premature rupture of membranes	
Mode of delivery (cesarean delivery)	
Child characteristics	
Preterm birth (≤36 weeks)	
Male sex	✓
Birth weight	
Neonatal jaundice with treatment	
Breastfeeding only	
Weight gain per day	
Presence of child's siblings	✓

© 2024 Fukuda S et al. *JAMA Network Open.*

eTable 5. List of Covariates Included in Model 3

Covariates	Included in the model
Maternal pre-pregnancy physical characteristics	
Height	
Weight before pregnancy	
Body mass index before pregnancy	
Maternal medical history	
Kawasaki disease	
Allergic disease	
Neurological/psychiatric disease	
Diabetes mellitus	
Cancer	
Paternal medical history	
Kawasaki disease	
Allergic disease	
Parental socioeconomic status	
Maternal education	✓
Paternal education	
Household income	✓
Spontaneous pregnancy	
Pregnancy complications	
Thyroid disease	
Diabetes mellitus	
Dietary intake during the second/third trimester	
Beans	
Vegetables	
Fruits	
Fermented foods	
Estimated folic acid from daily meals	

© 2024 Fukuda S et al. *JAMA Network Open.*

eTable 5. List of Covariates Included in Model 3 (continued)

Covariates	Included in the model
Supplementation during the first trimester (folic acid)	
Supplementation during the second/third trimester	
Folic acid	
Zinc	✓
Eicosapentaenoic acid	✓
Docosahexaenoic acid	✓
Lactobacillus-fermented beverages	✓
Lifestyle habits	
Smoking during pregnancy	
Drinking during pregnancy	
Maternal serum folic acid level during the second/third trimester	
Maternal age at birth	✓
Perinatal complications	
Threatened abortion/premature labor	
Gestational diabetes mellitus	
Gestational hypertension	
Premature rupture of membranes	
Mode of delivery (cesarean delivery)	
Child characteristics	
Preterm birth (≤36 weeks)	
Male sex	
Birth weight	
Neonatal jaundice with treatment	
Breastfeeding only	
Weight gain per day	
Presence of child's siblings	✓

eTable 6. Characteristics and Background of Participants Included and Excluded in the Analysis[a]

Variables	Included (N=87,702)	Excluded (N=16,360)	Standardized difference
Maternal pre-pregnancy physical characteristics			
Height, cm	158.1±5.3<br>158.0<br>(130.0–183.0)	157.9±5.4<br>158.0<br>(138.0–179.8)	0.04
Weight before pregnancy, kg	52.9±8.6<br>52.0 (29.0–127.6)	54.1±10.5<br>52.0 (32.0–130.0)	−0.12
BMI before pregnancy, kg/m^2	21.2±3.2<br>20.5 (13.2–52.8)	21.7±4.0<br>20.7 (13.3–49.8)	−0.15
Maternal medical history			
Kawasaki disease	378/87,372 (0.4)	52/13,425 (0.4)	0.01
Allergic disease	48,566/87,372 (55.6)	7121/13,425 (53.0)	0.05
Neurological/psychiatric disease	11,849/87,372 (13.6)	2006/13,425 (14.9)	−0.04
Diabetes mellitus	820/87,372 (0.9)	133/13,425 (1.0)	−0.01
Cancer	942/87,372 (1.1)	172/13,425 (1.3)	−0.02
Paternal medical history			
Kawasaki disease	198/45,581 (0.4)	17/5184 (0.3)	0.02
Allergic disease	21,196/45,581 (46.5)	2323/5184 (44.8)	0.03
Parental socioeconomic status			
Maternal education			
Beyond high school graduate	57,211/87,375 (65.5)	5477/11,046 (49.6)	0.33
Paternal education			
Beyond high school graduate	49,773/86,914 (57.3)	4919/10,879 (45.2)	0.24
Household income			
≥4 million JPY	49,800/81,914 (60.8)	5159/9996 (51.6)	0.19

© 2024 Fukuda S et al. *JAMA Network Open.*

eTable 6. Characteristics and Background of Participants Included and Excluded in the Analysis[a] (continued)

Variables	Included (N=87,702)	Excluded (N=16,360)	Standardized difference
Spontaneous pregnancy	81,312/87,678 (92.7)	14,643/15,793 (92.7)	0.00
Pregnancy complications			
Thyroid disease	1178/87,535 (1.3)	189/14,150 (1.3)	0.00
Diabetes mellitus	906/87,535 (1.0)	207/14,150 (1.5)	−0.04
Dietary intake during the second/third trimester			
Beans, g/day	54±76<br>34 (0–3960)	54±108<br>31 (0–5536)	0.00
Vegetables, g/day	89±92<br>66 (0–4705)	86±119<br>59 (0–4509)	0.03
Fruits, g/day	146±167<br>108 (0–9468)	145±235<br>98 (0–9927)	0.01
Fermented foods, g/day	122±158<br>84 (0–3171)	107±164<br>70 (0–4280)	0.09
Estimated folic acid from daily meals, μg/day	260±162<br>230 (3–9253)	252±197<br>154 (3–7091)	0.04

© 2024 Fukuda S et al. *JAMA Network Open.*

eTable 6. Characteristics and Background of Participants Included and Excluded in the Analysis[a] (continued)

Variables	Included (N=87,702)	Excluded (N=16,360)	Standardized difference
Supplementation during the second/third trimester			
Folic acid			
Daily	18,924/87,702 (21.6)	2052/10,894 (18.8)	0.07
At least once a week	12,351/87,702 (14.1)	1273/10,894 (11.7)	0.07
At least once a month	6535/87,702 (7.5)	752/10,894 (6.9)	0.02
Never	49,892/87,702 (56.9)	6817/10,894 (62.6)	−0.12
Zinc	3376/87,372 (3.9)	1074/10,962 (9.8)	−0.24
Eicosapentaenoic acid	988/87,257 (1.1)	437/10,943 (4.0)	−0.18
Docosahexaenoic acid	1937/87,307 (2.2)	828/10,953 (7.6)	−0.25
Lactobacillus-fermented beverages	46,188/87,497 (52.8)	5214/10,992 (47.4)	0.11
Lifestyle habits			
Smoking during pregnancy	3648/86,420 (4.2)	1902/11,099 (17.1)	−0.43
Drinking during pregnancy	9772/86,488 (11.3)	1341/11,133 (12.0)	−0.02
Maternal serum folic acid level during the second/third trimester, ng/mL	7.7±4.9<br>6.0 (0.7–20.0)	6.7±4.7<br>5.0 (0.6–20.0)	0.21
Maternal age at birth, years	31±5<br>31 (14–49)	30±6<br>30 (15–50)	0.22

© 2024 Fukuda S et al. *JAMA Network Open.*

eTable 6. Characteristics and Background of Participants Included and Excluded in the Analysis[a] (continued)

Variables	Included (N=87,702)	Excluded (N=16,360)	Standardized difference
Perinatal complications			
Threatened abortion/ premature labor	23,698/87,535 (27.1)	3966/14,150 (28.0)	−0.02
Gestational diabetes mellitus	2341/87,535 (2.7)	416/14,150 (2.9)	−0.02
Gestational hypertension	2713/87,535 (3.1)	515/14,150 (3.6)	−0.03
Premature rupture of membranes	7266/87,535 (8.3)	1137/14,150 (8.0)	0.01
Mode of delivery			
Cesarean delivery	17,164/87,330 (19.7)	2962/13,028 (22.7)	−0.08
Child characteristics			
Preterm birth (≤36 weeks)	4577/87,535 (5.2)	2546/14,084 (18.1)	−0.41
Male sex	44,998/87,702 (51.3)	6919/13,414 (24.3)	0.58
Birth weight, g	3014±423<br>3022 (398–5214)	2915±610<br>2992 (300–4906)	0.19
Neonatal jaundice with treatment	13,289/85,130 (15.6)	1956/12,003 (16.3)	−0.02
Breastfeeding only	37,183/87,289 (42.6)	4469/11,158 (40.1)	0.05
Weight gain per day, g/day	39±12<br>39 (−9–89)	38±12<br>38 (−3–89)	0.04
Presence of child's siblings	50,780/87,372 (58.1)	8464/13,469 (62.8)	−0.10

[a]Data are presented as n (%), mean ± standard deviation, or median (range).

BMI, body mass index.

© 2024 Fukuda S et al. *JAMA Network Open.*

eTable 7. Characteristics and Background of Participants (Maternal Serum Folic Acid Level ≥10 ng/mL or <10 ng/mL)[a]

Variables	Serum folic acid level ≥10 ng/mL (N=20,698)	Serum folic acid level <10 ng/mL (N=64,468)	Standardized difference
Maternal pre-pregnancy physical characteristics			
Height, cm	158.2±5.3<br>158.0<br>(137.0–181.0)	158.1±5.4<br>158.0<br>(130.0–183.0)	−0.01
Weight before pregnancy, kg	52.3±7.9<br>51.0 (31.0–117.0)	53.2±8.8<br>52.0 (29.0–125.0)	0.11
BMI before pregnancy, kg/m^2	20.9±2.9<br>20.3 (14.5–46.9)	21.3±3.3<br>20.6 (13.8–49.9)	0.13
Maternal medical history			
Kawasaki Disease	96/20,650 (0.5)	272/64,299 (0.4)	−0.01
Allergic disease	11,777/20,650<br>(57.0)	35,445/64,299<br>(55.1)	−0.04
Neurological/psychiatric disease	2999/20,650<br>(14.5)	8516/64,299<br>(13.2)	−0.04
Diabetes mellitus	220/20,650 (1.1)	565/64,299 (0.9)	−0.02
Cancer	243/20,650 (1.2)	658/64,299 (1.0)	−0.01
Paternal medical history			
Kawasaki Disease	47/11,358 (0.4)	145/33,495 (0.4)	0.00
Allergic disease	5438/11,358<br>(47.9)	15,406/33,495<br>(46.0)	−0.04

eTable 7. Characteristics and Background of Participants (Maternal Serum Folic Acid Level ≥10 ng/mL or <10 ng/mL)[a] (continued)

Variables	Serum folic acid level ≥10 ng/mL (N=20,698)	Serum folic acid level <10 ng/mL (N=64,468)	Standardized difference
Parental socioeconomic status			
Maternal education			
Beyond high school graduate	14,786/20,627 (71.7)	40,647/64,220 (63.3)	−0.18
Paternal education			
Beyond high school graduate	13,175/20,558 (64.1)	34,961/63,853 (54.8)	−0.19
Household income			
≥4 million JPY	12,991/19,485 (66.7)	35,228/60,055 (58.7)	−0.17
Spontaneous pregnancy	18,293/20,693 (88.4)	60,767/64,452 (94.3)	0.21
Pregnancy complications			
Thyroid disease	336/20,661 (1.6)	803/64,370 (1.2)	−0.03
Diabetes mellitus	253/20,661 (1.2)	628/64,370 (1.0)	−0.02
Dietary intake during the second/third trimester			
Beans, g/day	65±89<br>40 (0–2768)	51±70<br>32 (0–3960)	−0.18
Vegetables, g/day	103±103<br>77 (0–4705)	84±87<br>63 (0–2546)	−0.20
Fruits, g/day	152±165<br>114 (0–5869)	143±166<br>106 (0–9468)	−0.05
Fermented foods, g/day	148±179<br>112 (0–3171)	113±150<br>76 (0–3118)	−0.21
Estimated folic acid from daily meals, μg/day	271±161<br>242 (3–5759)	255±161<br>227 (3–9253)	−0.10

© 2024 Fukuda S et al. *JAMA Network Open.*

eTable 7. Characteristics and Background of Participants (Maternal Serum Folic Acid Level ≥10 ng/mL or <10 ng/mL)[a] (continued)

Variables	Serum folic acid level ≥10 ng/mL (N=20,698)	Serum folic acid level <10 ng/mL (N=64,468)	Standardized difference
Supplementation during the second/third trimester			
Folic acid			
Daily	12,401/20,698 (59.9)	5870/64,468 (9.1)	−1.26
At least once a week	4303/20,698 (20.8)	7672/64,468 (11.9)	−0.24
At least once a month	788/20,698 (3.8)	5579/64,468 (8.7)	0.20
Never	3206/20,698 (15.5)	45,347/64,468 (70.3)	1.33
Zinc	1458/20,573 (7.1)	1809/64,276 (2.8)	−0.20
Eicosapentaenoic acid	425/20,566 (2.1)	540/64,178 (0.8)	−0.10
Docosahexaenoic acid	908/20,566 (4.4)	968/64,217 (1.5)	−0.17
Lactobacillus-fermented beverages	11,902/20,633 (57.7)	32,980/64,335 (51.3)	−0.13
Lifestyle habits			
Smoking during pregnancy	512/20,447 (2.5)	3052/63,583 (4.8)	0.12
Drinking during pregnancy	2161/20,468 (10.6)	7317/63,630 (11.5)	0.03
Maternal serum folic acid level during the second/third trimester, ng/mL	15.1±3.5<br>14.5 (10.0–20.0)	5.3±2.0<br>5.0 (0.7–9.9)	−3.43
Maternal age at birth, years	32±5<br>32 (17–47)	31±5<br>31 (14–49)	−0.29

© 2024 Fukuda S et al. *JAMA Network Open.*

eTable 7. Characteristics and Background of Participants (Maternal Serum Folic Acid Level ≥10 ng/mL or <10 ng/mL)[a] (continued)

Variables	Serum folic acid level ≥10 ng/mL (N=20,698)	Serum folic acid level <10 ng/mL (N=64,468)	Standardized difference
Perinatal complications			
Threatened abortion/ premature labor	5687/20,661 (27.5)	17,475/64,370 (27.1)	−0.01
Gestational diabetes mellitus	615/20,661 (3.0)	1669/64,370 (2.6)	−0.02
Gestational hypertension	626/20,661 (3.0)	2014/64,370 (3.1)	0.01
Premature rupture of membranes	1924/20,661 (9.3)	5103/64,370 (7.9)	−0.05
Mode of delivery			
Cesarean delivery	4278/20,608 (20.8)	12,269/64,241 (19.1)	−0.04
Child characteristics			
Preterm birth (≤36 weeks)	1090/20,661 (5.3)	3164/64,370 (4.9)	−0.02
Male sex	10,671/20,698 (51.6)	33,039/64,468 (51.2)	−0.01
Birth weight, g	3000±423<br>3008 (512–4810)	3023±415<br>3030 (425–5214)	0.06
Neonatal jaundice with treatment	3338/20,029 (16.7)	9579/62,881 (15.2)	−0.04
Breastfeeding only	8212/20,615 (39.8)	28,032/64,175 (43.7)	0.08
Weight gain per day, g/day	38±12<br>38 (−9–88)	39±12<br>39 (−8–89)	0.05
Presence of child's siblings	9529/20,650 (46.1)	40,017/64,299 (62.2)	0.33

[a]Data are presented as n (%), mean ± standard deviation, or median (range).

BMI, body mass index.

© 2024 Fukuda S et al. *JAMA Network Open.*

eTable 8. Characteristics and Background of Participants (With and Without Maternal Folic Acid Supplementation During the First Trimester)[a]

Variables	Supplementation (+) (N=39,098)	Supplementation (−) (N=48,053)	Standardized difference
Maternal pre-pregnancy physical characteristics			
Height, cm	158.3±5.4<br>158.0 (130.0–183.0)	158.1±5.3<br>158.0 (137.0–180.0)	−0.04
Weight before pregnancy, kg	52.8±8.3<br>51.6 (31.0–127.6)	53.1±8.8<br>52.0 (29.0–125.0)	0.03
BMI before pregnancy, kg/m^2	21.1±3.1<br>20.4 (13.8–52.8)	21.2±3.3<br>20.5 (13.2–48.8)	0.05
Maternal medical history			
Kawasaki disease	169/39,098 (0.4)	209/48,053 (0.4)	0.00
Allergic disease	22,772/39,098 (58.2)	25,690/48,053 (53.5)	−0.10
Neurological/psychiatric disease	5764/39,098 (14.7)	6050/48,053 (12.6)	−0.06
Diabetes mellitus	355/39,098 (0.9)	460/48,053 (1.0)	0.01
Cancer	436/39,098 (1.1)	503/48,053 (1.0)	−0.01
Paternal medical history			
Kawasaki disease	94/21,317 (0.4)	103/24,097 (0.4)	0.00
Allergic disease	10,160/21,317 (47.7)	10,946/24,097 (45.4)	−0.04

© 2024 Fukuda S et al. *JAMA Network Open.*

eTable 8. Characteristics and Background of Participants (With and Without Maternal Folic Acid Supplementation During the First Trimester)[a] (continued)

Variables	Supplementation (+) (N=39,098)	Supplementation (−) (N=48,053)	Standardized difference
Parental socioeconomic status			
Maternal education			
Beyond high school graduate	27,479/38,962 (70.5)	29,391/47,864 (61.4)	−0.19
Paternal education			
Beyond high school graduate	24,411/38,830 (62.9)	25,051/47,546 (52.7)	−0.21
Household income			
≥4 million JPY	24,244/36,889 (65.7)	25,262/44,534 (56.7)	−0.19
Spontaneous pregnancy	35,159/39,098 (89.9)	45,647/48,051 (95.0)	0.19
Pregnancy complications			
Thyroid disease	582/39,016 (1.5)	591/47,968 (1.2)	−0.02
Diabetes mellitus	416/39,016 (1.1)	484/47,968 (1.0)	−0.01
Dietary intake during the second/third trimester			
Beans, g/day	58±77<br>36 (0–2768)	51±74<br>32 (0–3960)	−0.10
Vegetables, g/day	96±94<br>72 (0–4705)	83±89<br>61 (0–2546)	−0.14
Fruits, g/day	150±159<br>112 (0–5869)	142±174<br>104 (0–9468)	−0.04
Fermented foods, g/day	134±165<br>100 (0–3118)	111±151<br>75 (0–3171)	−0.15
Estimated folic acid from daily meals, μg/day	263±146<br>235 (3–4344)	257±169<br>227 (3–7334)	−0.04

© 2024 Fukuda S et al. *JAMA Network Open.*

eTable 8. Characteristics and Background of Participants (With and Without Maternal Folic Acid Supplementation During the First Trimester)[a] (continued)

Variables	Supplementation (+) (N=39,098)	Supplementation (−) (N=48,053)	Standardized difference
Supplementation during the second/third trimester			
Folic acid			
Daily	16,152/39,098 (41.3)	2,670/48,053 (5.6)	−0.93
At least once a week	9491/39,098 (24.3)	2786/48,053 (5.8)	−0.54
At least once a month	3591/39,098 (9.2)	2906/48,053 (6.0)	−0.12
Never	9864/39,098 (25.2)	39,691/48,053 (82.6)	1.41
Zinc	2475/38,914 (6.4)	872/47,909 (1.8)	−0.23
Eicosapentaenoic acid	657/38,867 (1.7)	325/47,842 (0.7)	−0.09
Docosahexaenoic acid	1338/38,890 (3.4)	587/47,869 (1.2)	−0.15
Lactobacillus-fermented beverages	22,415/38,996 (57.5)	23,497/47,954 (49.0)	−0.17
Lifestyle habits			
Smoking during pregnancy	1183/38,700 (3.1)	2429/47,495 (5.1)	0.10
Drinking during pregnancy	4079/38,737 (10.5)	5653/47,528 (11.9)	0.04
Maternal serum folic acid level during the second/third trimester, ng/mL	10.3±5.3<br>9.0 (1.2–20.0)	5.6±3.2<br>4.7 (0.7–20.0)	−1.06
Maternal age at birth, years	32±5<br>32 (14–49)	31±5<br>31 (15–48)	−0.18

© 2024 Fukuda S et al. *JAMA Network Open.*

eTable 8. Characteristics and Background of Participants (With and Without Maternal Folic Acid Supplementation During the First Trimester)[a] (continued)

Variables	Supplementation (+) (N=39,098)	Supplementation (−) (N=48,053)	Standardized difference
Perinatal complications			
Threatened abortion/ premature labor	10,889/39,016 (27.9)	12,703/47,968 (26.5)	−0.03
Gestational diabetes mellitus	1102/39,016 (2.8)	1224/47,968 (2.6)	−0.02
Gestational hypertension	1161/39,016 (3.0)	1537/47,968 (3.2)	0.01
Premature rupture of membranes	3484/39,016 (8.9)	3737/47,968 (7.8)	−0.04
Mode of delivery			
Cesarean delivery	7893/38,901 (20.3)	9168/47,880 (19.1)	−0.03
Child characteristics			
Preterm birth (≤36 weeks)	2097/39,016 (5.4)	2460/47,968 (5.1)	−0.01
Male sex	20,177/39,098 (51.6)	24,543/48,053 (51.1)	−0.01
Birth weight, g	3009±424<br>3018 (398–4890)	3017±422<br>3025 (425–5214)	0.02
Neonatal jaundice with treatment	6032/37,731 (16.0)	7181/46,860 (15.3)	−0.02
Breastfeeding only	15,962/38,937 (41.0)	21,009/47,812 (43.9)	0.06
Weight gain per day, g/day	39±12<br>39 (−9–89)	39±12<br>39 (−8–89)	0.03
Presence of child's siblings	19,929/39,098 (51.0)	30,717/48,053 (63.9)	0.26

[a]Data are presented as n (%), mean ± standard deviation, or median (range).

BMI, body mass index.

© 2024 Fukuda S et al. *JAMA Network Open.*

eTable 9. Characteristics and Background of Participants (With and Without Maternal Folic Acid Supplementation During the Second/Third Trimester)[a]

Variables	Supplementation (+) (N=31,275)	Supplementation (−) (N=56,427)	Standardized difference
Maternal pre-pregnancy physical characteristics			
Height, cm	158.2±5.4<br>158.0 (130.0–181.0)	158.1±5.3<br>158.0 (137.0–183.0)	−0.01
Weight before pregnancy, kg	52.7±8.3<br>51.0 (32.4–127.6)	53.1±8.7<br>52.0 (29.0–125.0)	0.05
BMI before pregnancy, kg/m^2	21.0±3.0<br>20.4 (13.2–52.8)	21.2±3.2<br>20.5 (13.8–48.8)	0.06
Maternal medical history			
Kawasaki disease	137/31,158 (0.4)	241/56,214 (0.4)	0.00
Allergic disease	17,963/31,158 (57.7)	30,603/56,214 (54.4)	−0.06
Neurological/psychiatric disease	4626/31,158 (14.8)	7223/56,214 (12.8)	−0.06
Diabetes mellitus	297/31,158 (1.0)	522/56,214 (0.9)	0.00
Cancer	369/31,158 (1.2)	573/56,214 (1.0)	−0.02
Paternal medical history			
Kawasaki disease	73/16,950 (0.4)	125/28,631 (0.4)	0.00
Allergic disease	8068/16,950 (47.6)	13,128/28,631 (45.9)	−0.04

© 2024 Fukuda S et al. *JAMA Network Open.*

eTable 9. Characteristics and Background of Participants (With and Without Maternal Folic Acid Supplementation During the Second/Third Trimester)[a] (continued)

Variables	Supplementation (+) (N=31,275)	Supplementation (−) (N=56,427)	Standardized difference
Parental socioeconomic status			
Maternal education			
Beyond high school graduate	21,799/31,162 (70.0)	35,412/56,213 (63.0)	−0.15
Paternal education			
Beyond high school graduate	19,378/31,036 (62.4)	30,395/55,878 (54.4)	−0.16
Household income			
≥4 million JPY	19,172/29,455 (65.1)	30,628/52,459 (58.4)	−0.14
Spontaneous pregnancy	28,197/31,264 (90.2)	53,115/56,414 (94.2)	0.15
Pregnancy complications			
Thyroid disease	459/31,206 (1.5)	719/56,329 (1.3)	−0.02
Diabetes mellitus	328/31,206 (1.1)	578/56,329 (1.0)	0.00
Dietary intake during the second/third trimester			
Beans, g/day	60±81<br>38 (0–2768)	51±73<br>32 (0–3960)	−0.11
Vegetables, g/day	99±97<br>74 (0–4705)	83±88<br>62 (0–2546)	−0.17
Fruits, g/day	151±165<br>114 (0–5869)	143±169<br>105 (0–9468)	−0.05
Fermented foods, g/day	137±166<br>104 (0–3171)	113±153<br>76 (0–2660)	−0.15
Estimated folic acid from daily meals, μg/day	266±156<br>238 (3–5759)	256±165<br>226 (3–9253)	−0.06

© 2024 Fukuda S et al. *JAMA Network Open.*

eTable 9. Characteristics and Background of Participants (With and Without Maternal Folic Acid Supplementation During the Second/Third Trimester)[a] (continued)

Variables	Supplementation (+) (N=31,275)	Supplementation (−) (N=56,427)	Standardized difference
Supplementation during the second/third trimester			
Folic acid			
Daily	18,924/31,275 (60.5)	0/56,427 (0.0)	-
At least once a week	12,351/31,275 (39.5)	0/56,427 (0.0)	-
At least once a month	0/31,275 (0.0)	6535/56,427 (11.6)	-
Never	0/31,275 (0.0)	49,892/56,427 (88.4)	-
Zinc	2720/31,084 (8.8)	656/56,288 (1.2)	−0.35
Eicosapentaenoic acid	712/31,035 (2.3)	276/56,222 (0.5)	−0.15
Docosahexaenoic acid	1440/31,058 (4.6)	497/56,249 (0.9)	−0.23
Lactobacillus-fermented beverages	18,388/31,175 (59.0)	27,800/56,322 (49.4)	−0.19
Lifestyle habits			
Smoking during pregnancy	1033/30,836 (3.3)	2615/55,584 (4.7)	0.07
Drinking during pregnancy	3328/30,885 (10.8)	6444/55,603 (11.6)	0.03
Maternal serum folic acid level during the second/third trimester, ng/mL	11.5±5.3<br>10.8 (1.2–20.0)	5.5±2.9<br>4.8 (0.7–20.0)	−1.40
Maternal age at birth, years	32±5<br>32 (14–49)	31±5<br>31 (15–48)	−0.21

© 2024 Fukuda S et al. *JAMA Network Open.*

eTable 9. Characteristics and Background of Participants (With and Without Maternal Folic Acid Supplementation During the Second/Third Trimester)[a] (continued)

Variables	Supplementation (+) (N=31,275)	Supplementation (−) (N=56,427)	Standardized difference
Perinatal complications			
Threatened abortion/ premature labor	8342/31,206 (26.7)	15,356/56,329 (27.3)	0.01
Gestational diabetes mellitus	854/31,206 (2.7)	1487/56,329 (2.6)	−0.01
Gestational hypertension	929/31,206 (3.0)	1784/56,329 (3.2)	0.01
Premature rupture of membranes	2762/31,206 (8.9)	4504/56,329 (8.0)	−0.03
Mode of delivery			
Cesarean delivery	6418/31,112 (20.6)	10,746/56,218 (19.1)	−0.04
Child characteristics			
Preterm birth (≤36 weeks)	1680/31,206 (5.4)	2897/56,329 (5.1)	−0.01
Male sex	16,134/31,275 (51.6)	28,864/56,427 (51.2)	−0.01
Birth weight, g	3008±428<br>3016 (398–4890)	3017±420<br>3025 (425–5214)	0.02
Neonatal jaundice with treatment	4935/30,185 (16.3)	8354/54,945 (15.2)	−0.03
Breastfeeding only	12,517/31,138 (40.2)	24,666/56,151 (43.9)	0.08
Weight gain per day, g/day	39±12<br>38 (−9–89)	39±12<br>39 (−8–89)	0.04
Presence of child's siblings	15,582/31,158 (50.0)	35,198/56,214 (62.6)	0.26

[a]Data are presented as n (%), mean ± standard deviation, or median (range).

BMI, body mass index.

© 2024 Fukuda S et al. *JAMA Network Open.*

eTable 10. Propensity Score Analysis of Maternal Serum Folic Acid Levels During the Second/Third Trimester and Onset of Kawasaki Disease[a]

Potential confounders	Exposures	Development of KD	Odds ratio	95% CI
Model 1[b]	Serum folic acid level <10 ng/mL	267/64,468 (0.41)	Reference	-
	≥10, <20 ng/mL	43/16,270 (0.26)	0.66	0.48–0.92
	≥20 ng/mL	13/4428 (0.29)	0.75	0.42–1.31
Model 2[c]	Serum folic acid level <10 ng/mL	267/64,468 (0.41)	Reference	-
	≥10, <20 ng/mL	43/16,270 (0.26)	0.66	0.48–0.92
	≥20 ng/mL	13/4428 (0.29)	0.74	0.42–1.29
Model 3[d]	Serum folic acid level <10 ng/mL	267/64,468 (0.41)	Reference	-
	≥10, <20 ng/mL	43/16,270 (0.26)	0.67	0.48–0.93
	≥20 ng/mL	13/4428 (0.29)	0.76	0.43–1.33

Abbreviations: KD, Kawasaki disease; CI, confidence interval.

a Data are presented as n (%).

b Details of covariates included in Model 1 are presented in eTable 1.

c Details of covariates included in Model 2 are presented in eTable 4.

d Details of covariates included in Model 3 are presented in eTable 5.

© 2024 Fukuda S et al. *JAMA Network Open.*

eFigure 1. Distribution of Propensity Scores Based on Model 1 According to Maternal Serum Folic Acid Levels

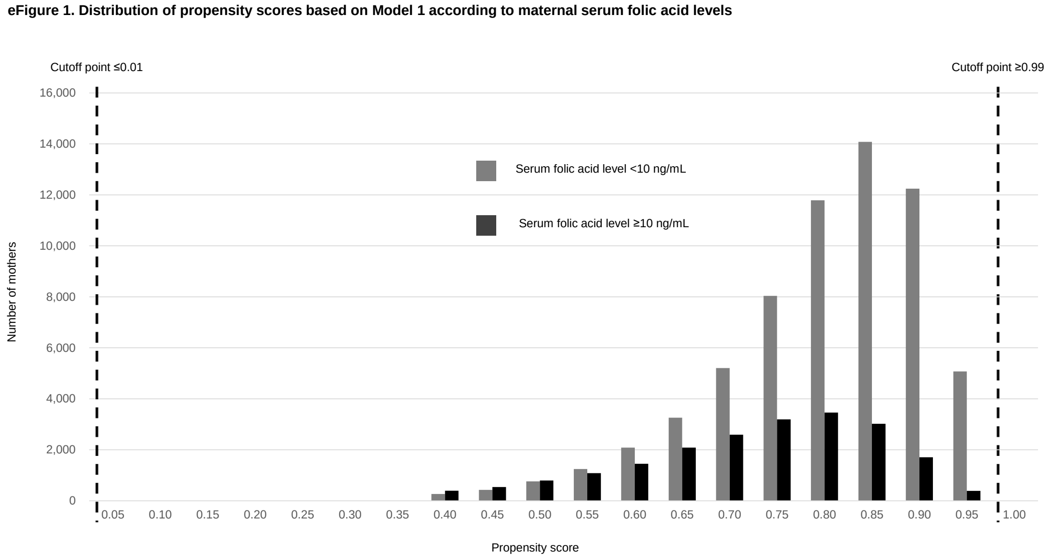

© 2024 Fukuda S et al. *JAMA Network Open.*

eFigure 2. Frequency of Folic Acid Supplementation According to Serum Folic Acid Level

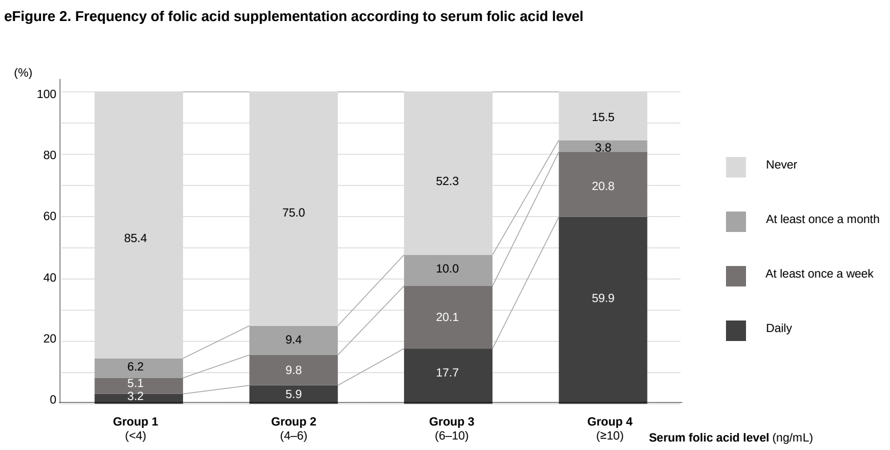

© 2024 Fukuda S et al. *JAMA Network Open.*
