# Supplementary material for: Maternal Serum Folic Acid Levels and Onset of Kawasaki Disease in Offspring During Infancy
Source: JAMA Netw Open. 2023 Dec 28;6(12):e2349942. doi: 10.1001/jamanetworkopen.2023.49942 (PMC10755611; doi:10.1001/jamanetworkopen.2023.49942)
Supplement: Supplement 2. — Japan Environment and Children’s Study Group Members [file jamanetwopen-e2349942-s002.pdf]

*First name, last name, and suffix (if applicable) are required and will appear in PubMed.

*Group Name(s): Japan Environment and Children's Study Group

***First Name and Middle Initial(s)**	***Last Name**	***Suffix (eg, Jr, III)**	Academic Degrees	Institution	Location (city, state/province, country)	Role or Contribution, eg, chair, principal investigator	Group (if more than 1 Group listed in the byline) and/or Subgroup (eg, Steering Committee)
Michihiro	Kamijima		MD, PhD	Nagoya City University	Nagoya, Japan	Principal investigator	Aichi Regional Centre
Shin	Yamazaki		DrPH	National Institute for Environmental Studies	Tsukuba, Japan		Programme Office
Yukihiro	Ohya		MD, PhD	National Center for Child Health and Development	Tokyo, Japan		Medical Support Centre
Reiko	Kishi		MD, PhD, MPH	Hokkaido University	Sapporo, Japan		Hokkaido Regional Centre
Nobuo	Yaegashi		MD, PhD	Tohoku University	Sendai, Japan		Miyagi Regional Center
Koichi	Hashimoto		MD, PhD	Fukushima Medical University	Fukushima, Japan		Fukushima Regional Center
Chisato	Mori		MD, PhD	Chiba University	Chiba, Japan		Chiba Regional Centre
Shuichi	Ito		MD, PhD	Yokohama City University	Yokohama, Japan		Kanagawa Regional Centre
Zentaro	Yamagata		MD, PhD	University of Yamanashi	Chuo, Japan		Koshin Regional Centre
Hidekuni	Inadera		MD, PhD	University of Toyama	Toyama, Japan		Toyama Regional Centre
Takeo	Nakayama		MD, PhD	Kyoto University	Kyoto, Japan		Kyoto Regional Centre
Tomotaka	Sobue		MD, PhD, MPH	Osaka University	Suita, Japan		Osaka Regional Centre
Masayuki	Shima		MD, PhD	Hyogo Medical University	Nishinomiya, Japan		Hyogo Regional Centre
Seiji	Kageyama		MD, PhD	Tottori University	Yonago, Japan		Tottori Regional Centre
Narufumi	Suganuma		MD, PhD	Kochi University	Nankoku, Japan		Kochi Regional Centre
Shoichi	Ohga		MD, PhD	Kyushu University	Fukuoka, Japan		Fukuoka Regional Centre
Takahiko	Katoh		MD, PhD	Kumamoto University	Kumamoto, Japan		South Kyusyu/Okinawa Regional Centre
