# Supplementary material for: Maternal Serum Folic Acid Levels and Onset of Kawasaki Disease in Offspring During Infancy
Source: JAMA Netw Open. 2023 Dec 28;6(12):e2349942. doi: 10.1001/jamanetworkopen.2023.49942 (PMC10755611; doi:10.1001/jamanetworkopen.2023.49942)
Supplement: Supplement 3. — Data Sharing Statement [file jamanetwopen-e2349942-s003.pdf]

Data Sharing Statement

Fukuda. Maternal Serum Folic Acid Levels and Onset of Kawasaki Disease in Offspring During Infancy. *JAMA Netw Open*. Published January 03, 2024.
doi:10.1001/jamanetworkopen.2023.49942

Data
Data available: No

Additional Information
Explanation for why data not available: The Japanese Ministry of the Environment, which is leading this study, has not authorized the release of the data set at this time. There is a possibility that the data may be made public in the future.
